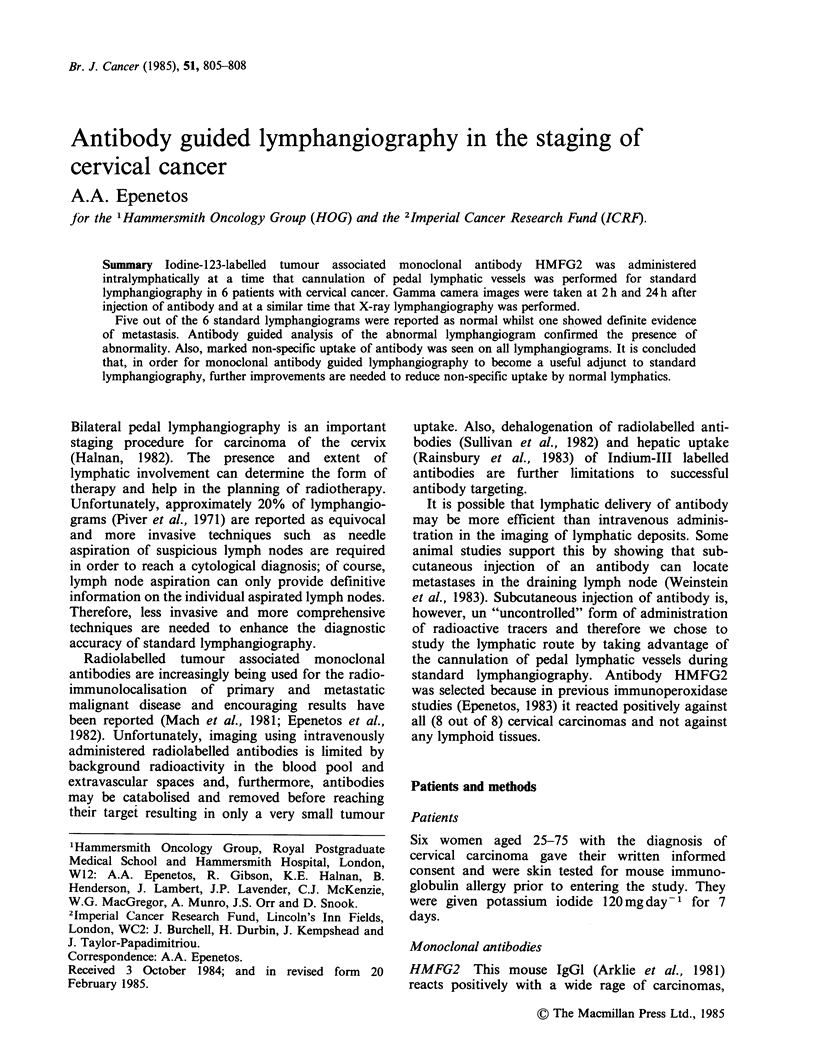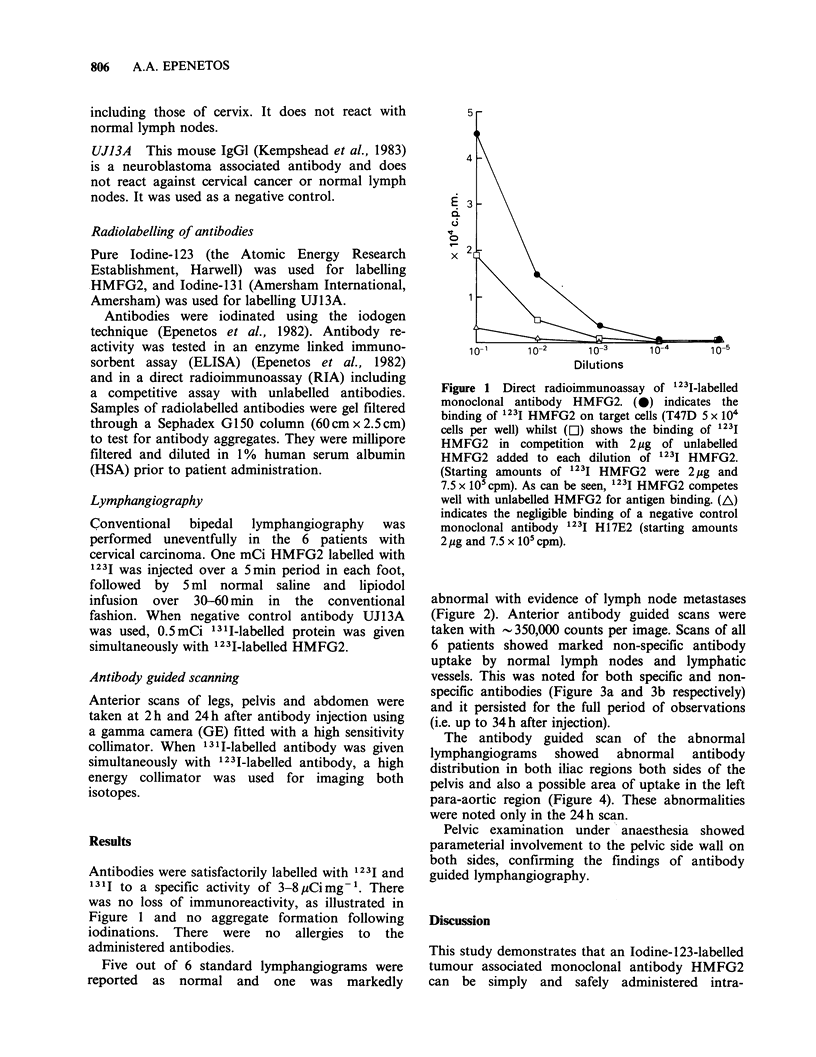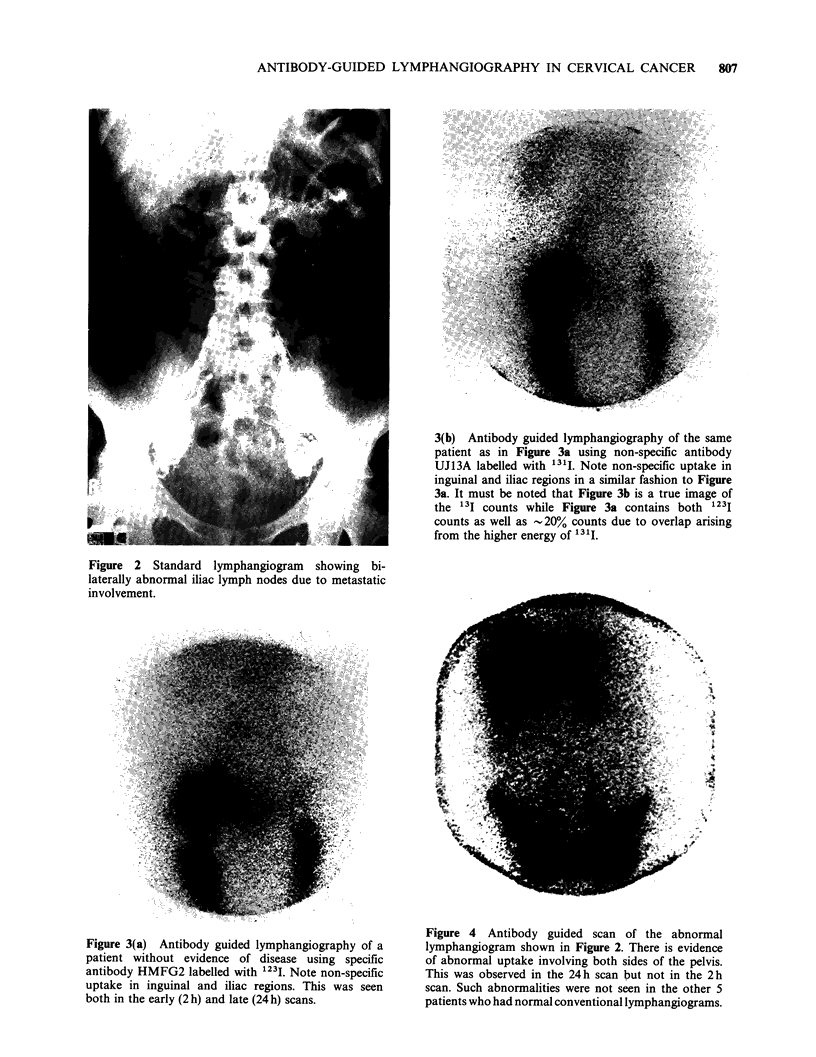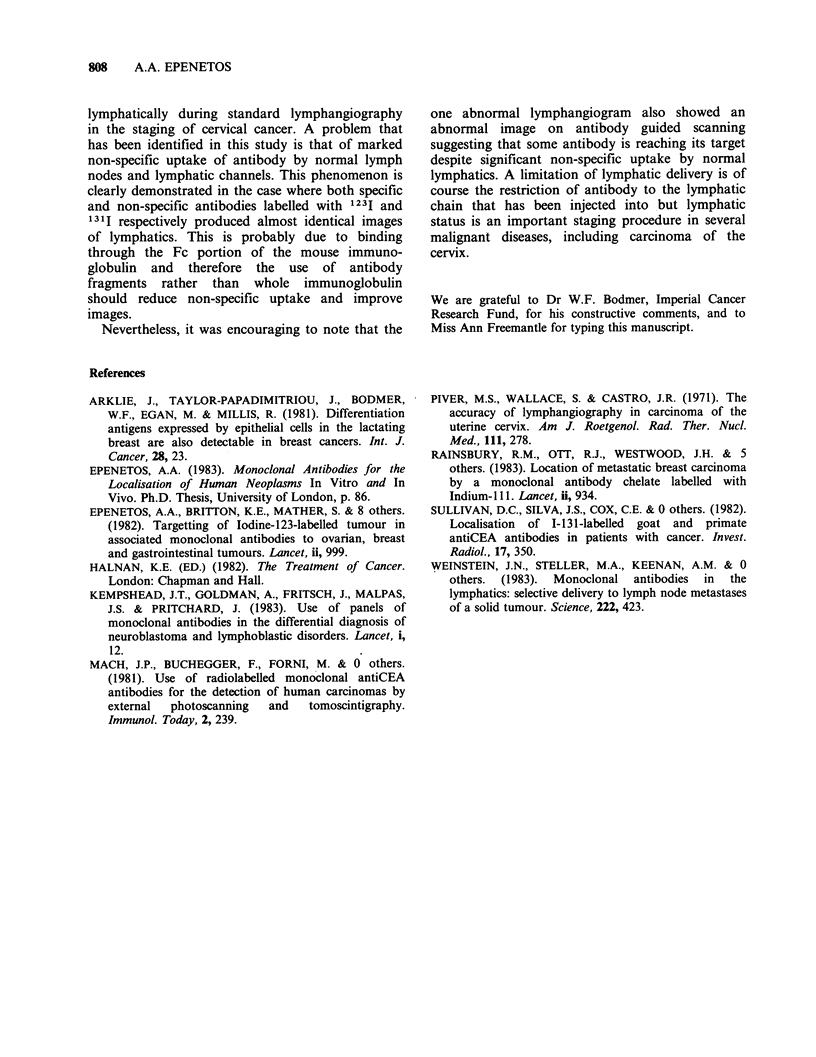# Antibody guided lymphangiography in the staging of cervical cancer.

**DOI:** 10.1038/bjc.1985.125

**Published:** 1985-06

**Authors:** A. A. Epenetos

## Abstract

**Images:**


*Br. J. Cancer* (1985), **51**, 805–808

### Antibody guided lymphangiography in the staging of cervical cancer

A.A. Epenetos

*for the* [1]*Hammersmith Oncology Group (HOG) and the* [2]*Imperial Cancer Research Fund (ICRF).*

**Summary** Iodine-123-labelled tumour associated monoclonal antibody HMFG2 was administered intralymphatically at a time that cannulation of pedal lymphatic vessels was performed for standard lymphangiography in 6 patients with cervical cancer. Gamma camera images were taken at 2 h and 24 h after injection of antibody and at a similar time that X-ray lymphangiography was performed.

Five out of the 6 standard lymphangiograms were reported as normal whilst one showed definite evidence of metastasis. Antibody guided analysis of the abnormal lymphangiogram confirmed the presence of abnormality. Also, marked non-specific uptake of antibody was seen on all lymphangiograms. It is concluded that, in order for monoclonal antibody guided lymphangiography to become a useful adjunct to standard lymphangiography, further improvements are needed to reduce non-specific uptake by normal lymphatics.

Bilateral pedal lymphangiography is an important staging procedure for carcinoma of the cervix (Halnan, 1982). The presence and extent of lymphatic involvement can determine the form of therapy and help in the planning of radiotherapy. Unfortunately, approximately 20% of lymphangiograms (Piver *et al.*, 1971) are reported as equivocal and more invasive techniques such as needle aspiration of suspicious lymph nodes are required in order to reach a cytological diagnosis; of course, lymph node aspiration can only provide definitive information on the individual aspirated lymph nodes. Therefore, less invasive and more comprehensive techniques are needed to enhance the diagnostic accuracy of standard lymphangiography.

Radiolabelled tumour associated monoclonal antibodies are increasingly being used for the radioimmunolocalisation of primary and metastatic malignant disease and encouraging results have been reported (Mach *et al.*, 1981; Epenetos *et al.*, 1982). Unfortunately, imaging using intravenously administered radiolabelled antibodies is limited by background radioactivity in the blood pool and extravascular spaces and, furthermore, antibodies may be catabolised and removed before reaching their target resulting in only a very small tumour uptake. Also, dehalogenation of radiolabelled antibodies (Sullivan *et al.*, 1982) and hepatic uptake (Rainsbury *et al.*, 1983) of Indium-III labelled antibodies are further limitations to successful antibody targeting.

It is possible that lymphatic delivery of antibody may be more efficient than intravenous administration in the imaging of lymphatic deposits. Some animal studies support this by showing that subcutaneous injection of an antibody can locate metastases in the draining lymph node (Weinstein *et al.*, 1983). Subcutaneous injection of antibody is, however, un "uncontrolled" form of administration of radioactive tracers and therefore we chose to study the lymphatic route by taking advantage of the cannulation of pedal lymphatic vessels during standard lymphangiography. Antibody HMFG2 was selected because in previous immunoperoxidase studies (Epenetos, 1983) it reacted positively against all (8 out of 8) cervical carcinomas and not against any lymphoid tissues.

### Patients and methods

### *Patients*

Six women aged 25–75 with the diagnosis of cervical carcinoma gave their written informed consent and were skin tested for mouse immunoglobulin allergy prior to entering the study. They were given potassium iodide 120 mg day$^{-1}$ for 7 days.

### *Monoclonal antibodies*

*HMFG2* This mouse IgGl (Arklie *et al.*, 1981) reacts positively with a wide rage of carcinomas,

[1]Hammersmith Oncology Group, Royal Postgraduate Medical School and Hammersmith Hospital, London, W12: A.A. Epenetos, R. Gibson, K.E. Halnan, B. Henderson, J. Lambert, J.P. Lavender, C.J. McKenzie, W.G. MacGregor, A. Munro, J.S. Orr and D. Snook.
[2]Imperial Cancer Research Fund, Lincoln's Inn Fields, London, WC2: J. Burchell, H. Durbin, J. Kempshead and J. Taylor-Papadimitriou.
Correspondence: A.A. Epenetos.
Received 3 October 1984; and in revised form 20 February 1985.

© The Macmillan Press Ltd., 1985

including those of cervix. It does not react with normal lymph nodes.

*UJ13A* This mouse IgGl (Kempshead *et al.*, 1983) is a neuroblastoma associated antibody and does not react against cervical cancer or normal lymph nodes. It was used as a negative control.

*Radiolabelling of antibodies*

Pure Iodine-123 (the Atomic Energy Research Establishment, Harwell) was used for labelling HMFG2, and Iodine-131 (Amersham International, Amersham) was used for labelling UJ13A.

Antibodies were iodinated using the iodogen technique (Epenetos *et al.*, 1982). Antibody reactivity was tested in an enzyme linked immunosorbent assay (ELISA) (Epenetos *et al.*, 1982) and in a direct radioimmunoassay (RIA) including a competitive assay with unlabelled antibodies. Samples of radiolabelled antibodies were gel filtered through a Sephadex G150 column (60 cm x 2.5 cm) to test for antibody aggregates. They were millipore filtered and diluted in 1% human serum albumin (HSA) prior to patient administration.

*Lymphangiography*

Conventional bipedal lymphangiography was performed uneventfully in the 6 patients with cervical carcinoma. One mCi HMFG2 labelled with $^{123}$I was injected over a 5 min period in each foot, followed by 5 ml normal saline and lipiodol infusion over 30–60 min in the conventional fashion. When negative control antibody UJ13A was used, 0.5 mCi $^{131}$I-labelled protein was given simultaneously with $^{123}$I-labelled HMFG2.

*Antibody guided scanning*

Anterior scans of legs, pelvis and abdomen were taken at 2 h and 24 h after antibody injection using a gamma camera (GE) fitted with a high sensitivity collimator. When $^{131}$I-labelled antibody was given simultaneously with $^{123}$I-labelled antibody, a high energy collimator was used for imaging both isotopes.

### Results

Antibodies were satisfactorily labelled with $^{123}$I and $^{131}$I to a specific activity of 3–8 $\mu$Ci mg$^{-1}$. There was no loss of immunoreactivity, as illustrated in Figure 1 and no aggregate formation following iodinations. There were no allergies to the administered antibodies.

Five out of 6 standard lymphangiograms were reported as normal and one was markedly

**Figure 1** Direct radioimmunoassay of $^{123}$I-labelled monoclonal antibody HMFG2. (●) indicates the binding of $^{123}$I HMFG2 on target cells (T47D $5 \times 10^4$ cells per well) whilst (□) shows the binding of $^{123}$I HMFG2 in competition with 2 $\mu$g of unlabelled HMFG2 added to each dilution of $^{123}$I HMFG2. (Starting amounts of $^{123}$I HMFG2 were 2 $\mu$g and $7.5 \times 10^5$ cpm). As can be seen, $^{123}$I HMFG2 competes well with unlabelled HMFG2 for antigen binding. (△) indicates the negligible binding of a negative control monoclonal antibody $^{123}$I H17E2 (starting amounts 2 $\mu$g and $7.5 \times 10^5$ cpm).

abnormal with evidence of lymph node metastases (Figure 2). Anterior antibody guided scans were taken with ~350,000 counts per image. Scans of all 6 patients showed marked non-specific antibody uptake by normal lymph nodes and lymphatic vessels. This was noted for both specific and non-specific antibodies (Figure 3a and 3b respectively) and it persisted for the full period of observations (i.e. up to 34 h after injection).

The antibody guided scan of the abnormal lymphangiograms showed abnormal antibody distribution in both iliac regions both sides of the pelvis and also a possible area of uptake in the left para-aortic region (Figure 4). These abnormalities were noted only in the 24 h scan.

Pelvic examination under anaesthesia showed parameterial involvement to the pelvic side wall on both sides, confirming the findings of antibody guided lymphangiography.

### Discussion

This study demonstrates that an Iodine-123-labelled tumour associated monoclonal antibody HMFG2 can be simply and safely administered intra-

**Figure 2** Standard lymphangiogram showing bilaterally abnormal iliac lymph nodes due to metastatic involvement.

**Figure 3(a)** Antibody guided lymphangiography of a patient without evidence of disease using specific antibody HMFG2 labelled with $^{123}$I. Note non-specific uptake in inguinal and iliac regions. This was seen both in the early (2 h) and late (24 h) scans.

**3(b)** Antibody guided lymphangiography of the same patient as in **Figure 3a** using non-specific antibody UJ13A labelled with $^{131}$I. Note non-specific uptake in inguinal and iliac regions in a similar fashion to **Figure 3a**. It must be noted that **Figure 3b** is a true image of the $^{13}$I counts while **Figure 3a** contains both $^{123}$I counts as well as $\sim$20% counts due to overlap arising from the higher energy of $^{131}$I.

**Figure 4** Antibody guided scan of the abnormal lymphangiogram shown in **Figure 2**. There is evidence of abnormal uptake involving both sides of the pelvis. This was observed in the 24 h scan but not in the 2 h scan. Such abnormalities were not seen in the other 5 patients who had normal conventional lymphangiograms.

lymphatically during standard lymphangiography in the staging of cervical cancer. A problem that has been identified in this study is that of marked non-specific uptake of antibody by normal lymph nodes and lymphatic channels. This phenomenon is clearly demonstrated in the case where both specific and non-specific antibodies labelled with $^{123}I$ and $^{131}I$ respectively produced almost identical images of lymphatics. This is probably due to binding through the Fc portion of the mouse immunoglobulin and therefore the use of antibody fragments rather than whole immunoglobulin should reduce non-specific uptake and improve images.

Nevertheless, it was encouraging to note that the one abnormal lymphangiogram also showed an abnormal image on antibody guided scanning suggesting that some antibody is reaching its target despite significant non-specific uptake by normal lymphatics. A limitation of lymphatic delivery is of course the restriction of antibody to the lymphatic chain that has been injected into but lymphatic status is an important staging procedure in several malignant diseases, including carcinoma of the cervix.

We are grateful to Dr W.F. Bodmer, Imperial Cancer Research Fund, for his constructive comments, and to Miss Ann Freemantle for typing this manuscript.

### References

ARKLIE, J., TAYLOR-PAPADIMITRIOU, J., BODMER, W.F., EGAN, M. & MILLIS, R. (1981). Differentiation antigens expressed by epithelial cells in the lactating breast are also detectable in breast cancers. *Int. J. Cancer*, **28**, 23.

EPENETOS, A.A. (1983). *Monoclonal Antibodies for the Localisation of Human Neoplasms* In Vitro *and* In Vivo. Ph.D. Thesis, University of London, p. 86.

EPENETOS, A.A., BRITTON, K.E., MATHER, S. & 8 others. (1982). Targetting of Iodine-123-labelled tumour in associated monoclonal antibodies to ovarian, breast and gastrointestinal tumours. *Lancet*, **ii**, 999.

HALNAN, K.E. (ED.) (1982). *The Treatment of Cancer*. London: Chapman and Hall.

KEMPSHEAD, J.T., GOLDMAN, A., FRITSCH, J., MALPAS, J.S. & PRITCHARD, J. (1983). Use of panels of monoclonal antibodies in the differential diagnosis of neuroblastoma and lymphoblastic disorders. *Lancet*, **i**, 12.

MACH, J.P., BUCHEGGER, F., FORNI, M. & 0 others. (1981). Use of radiolabelled monoclonal antiCEA antibodies for the detection of human carcinomas by external photoscanning and tomoscintigraphy. *Immunol. Today*, **2**, 239.

PIVER, M.S., WALLACE, S. & CASTRO, J.R. (1971). The accuracy of lymphangiography in carcinoma of the uterine cervix. *Am J. Roetgenol. Rad. Ther. Nucl. Med.*, **111**, 278.

RAINSBURY, R.M., OTT, R.J., WESTWOOD, J.H. & 5 others. (1983). Location of metastatic breast carcinoma by a monoclonal antibody chelate labelled with Indium-111. *Lancet*, **ii**, 934.

SULLIVAN, D.C., SILVA, J.S., COX, C.E. & 0 others. (1982). Localisation of I-131-labelled goat and primate antiCEA antibodies in patients with cancer. *Invest. Radiol.*, **17**, 350.

WEINSTEIN, J.N., STELLER, M.A., KEENAN, A.M. & 0 others. (1983). Monoclonal antibodies in the lymphatics: selective delivery to lymph node metastases of a solid tumour. *Science*, **222**, 423.